# The Presence of Small Nerve Fibers in the Tumor Microenvironment as Predictive Biomarker of Oncological Outcome Following Partial Hepatectomy for Intrahepatic Cholangiocarcinoma

**DOI:** 10.3390/cancers13153661

**Published:** 2021-07-21

**Authors:** Jan Bednarsch, Xiuxiang Tan, Zoltan Czigany, Dong Liu, Sven Arke Lang, Shivan Sivakumar, Jakob Nikolas Kather, Simone Appinger, Mika Rosin, Shiva Boroojerdi, Edgar Dahl, Nadine Therese Gaisa, Marcel den Dulk, Mariëlle Coolsen, Tom Florian Ulmer, Ulf Peter Neumann, Lara Rosaline Heij

**Affiliations:** 1 Department of Surgery and Transplantation, University Hospital RWTH Aachen, 52074 Aachen, Germany; jbednarsch@ukaachen.de (J.B.); x.tan@maastrichtuniversity.nl (X.T.); zczigany@ukaachen.de (Z.C.); dliu@ukaachen.de (D.L.); svlang@ukaachen.de (S.A.L.); sappinger@ukaachen.de (S.A.); mrosin@ukaachen.de (M.R.); shiboroojerd@ukaachen.de (S.B.); fulmer@ukaachen.de (T.F.U.); uneumann@ukaachen.de (U.P.N.); 2 NUTRIM School of Nutrition and Translational Research in Metabolism, Maastricht University, 6211 LK Maastricht, The Netherlands; 3 Department of Oncology, University of Oxford, Oxford OX3 7DQ, UK; shivan.sivakumar@oncology.ox.ac.uk; 4 Kennedy Institute of Rheumatology, University of Oxford, Oxford OX3 7FY, UK; 5 Department of Medicine III, University Hospital RWTH Aachen, 52074 Aachen, Germany; jkather@ukaachen.de; 6 Institute of Pathology, University Hospital RWTH Aachen, 52074 Aachen, Germany; edahl@ukaachen.de (E.D.); ngaisa@ukaachen.de (N.T.G.); 7 Department of Surgery, Maastricht University Medical Center (MUMC), 6229 HX Maastricht, The Netherlands; marcel.den.dulk@mumc.nl (M.d.D.); Marielle.coolsen@mumc.nl (M.C.)

**Keywords:** intrahepatic cholangiocarcinoma, nerve fibers, tumor microenvironment, oncological outcome, biomarker

## Abstract

**Simple Summary:**

Nerve fibers in the microenvironment of malignant tumors have been shown to be an important prognostic factor for long-term survival in various cancer types; however, their role in intrahepatic cholangiocarcinoma remains to be determined. Therefore, the impact of nerve fibers on long-term survival was investigated in a large European cohort of patients with intrahepatic cholangiocarcinoma who were treated by curative-intent surgical resection. By univariate and multivariate statistics, the absence of nerve fibers was determined to be an independent predictor of impaired long-term survival. A group comparison between patients with and without nerve fibers showed a statically significant difference with a cancer-specific 5-year-survival of 47% in patients with nerve fibers compared to 21% in patients without nerve fibers. Thus, the presence of nerve fibers in the microenvironment of intrahepatic cholangiocarcinoma is revealed as a novel and important prognostic biomarker in these patients.

**Abstract:**

The oncological role of the density of nerve fibers (NFs) in the tumor microenvironment (TME) in intrahepatic cholangiocarcinoma (iCCA) remains to be determined. Therefore, data of 95 iCCA patients who underwent hepatectomy between 2010 and 2019 was analyzed regarding NFs and long-term outcome. Extensive group comparisons were carried out and the association of cancer-specific survival (CSS) and recurrence-free survival (RFS) with NFs were assessed using Cox regression models. Patients with iCCA and NFs showed a median CSS of 51 months (5-year-CSS = 47%) compared to 27 months (5-year-CSS = 21%) in patients without NFs (*p* = 0.043 log rank). Further, NFs (hazard ratio (HR) = 0.39, *p* = 0.002) and N-category (HR = 2.36, *p* = 0.010) were identified as independent predictors of CSS. Patients with NFs and without nodal metastases displayed a mean CSS of 89 months (5-year-CSS = 62%), while patients without NFs or with nodal metastases but not both showed a median CCS of 27 months (5-year-CSS = 25%) and patients with both positive lymph nodes and without NFs showed a median CCS of 10 months (5-year-CSS = 0%, *p* = 0.001 log rank). NFs in the TME are, therefore, a novel and important prognostic biomarker in iCCA patients. NFs alone and in combination with nodal status is suitable to identify iCCA patients at risk of poor oncological outcomes following curative-intent surgery.

## 1. Introduction

Based on the anatomical localization of the primary tumor, cholangiocellular carcinomas (CCA) can be devided into intrahepatic CCA (iCCA), perihilar CCA (pCCA) and distal CCA (dCCA) [1]. Even though iCCA is the least common subtype of CCA, it still comprises about 15% of all primary liver tumors [2]. The ever-increasing global incidence of iCCA underlines the oncological significance of this disease [3,4]. Although, in some cases, the etiology of CCA remains unclear, cholestatic conditions and diseases associated with chronic inflammation are considered to be the major traditional risk factors in the oncogenesis of CCA [5]. In iCCA patients in particular, etiological factors such as parenchymal disease related to hepatic cirrhosis, viral hepatis or chronic alcohol consumption may play a pronounced role, illustrating distinct differences compared to the extrahepatic subtypes of CCA [5]. Irrespective of the CCA subentity, radical surgery with lymphadenectomy followed by adjuvant chemotherapy is considered as the current gold-standard approach as it provides improved long-term outcomes in comparison to merely medical or interventional treatment [6,7,8].

Radical resection of iCCA often requires extended liver resections as iCCA is often diagnosed in advanced disease stages. This may often result in increased perioperative morbidity and mortality [9]. Over the past decades, new surgical techniques have entered the clinical arena (e.g., Associating Liver Partition and Portal Vein Ligation for Staged Hepatectomy (ALPPS), preoperative portal vein embolization (PVE)), allowing surgery in patients even with a large tumor burden. Furthermore, as modern perioperative management facilitated surgery in elderly or patients with multiple comorbidities, more patients become candidates for radical surgical therapy [7,10,11]. Despite these advancements, the overall oncological prognosis in iCCA remains poor even after “curative-intent” surgery, with early tumor recurrence in many patients [12,13,14,15]. Identifying patients with particularly favorable oncological prognosis may allow individualized post-resection surveillance and therapy and is, therefore, of upmost clinical and scientific importance.

**Figure 1:**
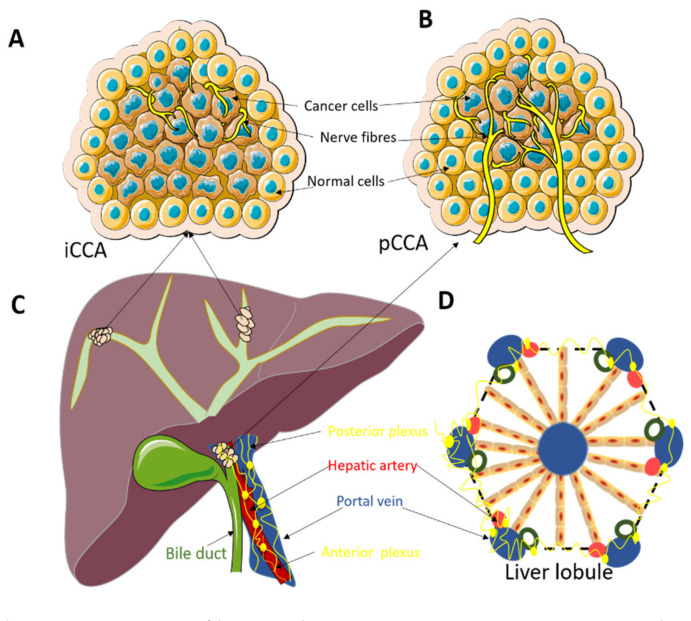
(**A**): Nerve fibers in the tumor microenvironment in intrahepatic cholangiocarcinoma (iCCA). The small nerve fibers are mainly distributed at the edge of the tumor. (**B**): Nerve fibers in the tumor microenvironment in perihilar cholangiocarcinoma (pCCA). The small nerve fibers are growing in between the tumor glands and not just at the edge of the tumor as in iCCA. (**C**): Localization of iCCA and pCCA tumors. ICCA is located centrally in the liver and usually presents with a big tumor mass. PCCA are located at the liver hilum and usually present as smaller tumors. (**D**): Architecture of the normal liver lobule. The normal innervation of the liver lobule can possibly explain the observation of the presence of the small nerve fibers at the edge of the tumor in iCCA and the pattern of mixed distribution of small nerve fibers in pCCA.

**Figure 2:**
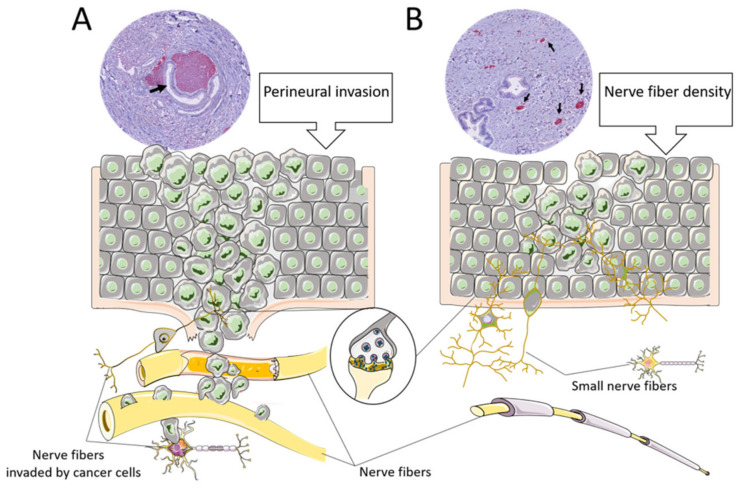
The difference between nerve fiber density and perineural invasion (PNI). (**A**): Graphical overview of perineural invasion. The cancer cells invade the endoneurial or epineurial sheeth of a larger nerve fiber. The immunohistochemical PGP9.5 staining illustrates the nerve fiber with cancer tissue around it. (**B**): Graphical overview of nerve fiber density. The small nerve fibers grow in the tumor microenvironment but are usually not invaded by cancer cells. The immunohistochemical PGP9.5 staining shows small positive (red) dots.

Our group has recently reported the significant prognostic value of nerve fibers (NFs) in the tumor microenvironment (TME) in a cohort pCCA patients [16] (Figure 1). CCAs often show perineural invasion (PNI), which can be recognized on routine hematoxylin and eosin (H&E) staining. Nevertheless, there is an important difference between traditional PNI and NFs concerning the size of the nerve fibers. The nerve fibers included in the NF count have a smaller diameter and are usually not visible on H&E routine staining and require additional immunohistochemical staining (Figure 2). Although NFs, as prognostic biomarkers, have also been investigated not just in pCCA but also in other malignancies, e.g., colorectal or gastric cancer and pancreatic ductal adenocarcinoma (PDAC) [17,18,19,20], their role in iCCA remains to be determined. Therefore, in the present study, we aimed to investigate NFs as a prognostic marker in a European cohort of iCCA patients undergoing curative-intent surgery.

## 2. Materials and Methods

### 2.1. Patient Cohort

All consecutive patients scheduled for surgical resection for iCCA at the University Hospital RWTH Aachen (UH-RWTH) between 2010 and 2019 were considered for inclusion in this study. Out of the complete cohort of patients (*n* = 120), a subset of individuals (*n* = 24) were excluded (*n* = 10 cases of perioperative mortality; *n* = 14 with missing NF data). Subsequently, a final cohort of 96 patients was analyzed. The study was evaluated and approved by the Institutional Review Board of the Medical Faculty of the RWTH-Aachen University (EK 106/18) and conducted in accordance with the principles of the Declaration of Helsinki, and good clinical practice guidelines (ICH-GCP).

### 2.2. Oncological Staging and Surgical Technique

All patients included in this study underwent a detailed clinical work-up as previously described [7,15,16]. Briefly, resection planning was carried out, and the presence of distant metastases was ruled out using magnetic resonance imaging (MRI) and/or multiphase computed tomography imaging (CT). Further, the preoperative work-up comprised liver volumetry and portal vein embolization (PVE) in patients with insufficient future liver remnant (FLR) scheduled for right-sided hepatectomy. The indication for surgical resection as the primary treatment was based on the final clinical evaluation by one of the senior hepatobiliary staff surgeons and approved by the local multidisciplinary tumor board in all cases. Surgery was carried out as previously described [7,15]. Depending on the local tumor extent, surgical procedures ranged from limited atypical resections to extended liver resections as well as vascular resections and reconstructions in cases with tumors extending to the liver hilum and ALPPS or PVE in individuals with insufficient FLR (Table 1). A systematic lymphadenectomy including the celiac, the posterior pancreaticoduodenal, the common hepatic, the periportal and pericholedochal lymph nodes was routinely carried out in all cases. All specimens were routinely evaluated by a trained pathologist.

### 2.3. Adjuvant Therapy and Patient Follow-Up

Adjuvant therapy was advised by the multidisciplinary tumor board for patients diagnosed with high-risk characteristics (e.g., R1 resection or positive nodal status) from 2010 to 2017. From 2017 on, adjuvant therapy was recommended in every case in accordance with findings of the BILCAP (Capecitabine compared with observation in resected biliary tract cancer) trial [8]. Each patient underwent a regular follow-up by the referring oncologist or the local outpatient clinic including standard laboratory blood tests with tumor markers (carbohydrate antigen (CA) 19–9), clinical examinations and cross-sectional imaging. Additional diagnostics, e.g., imaging and/or biopsy, were performed if tumor recurrence was suspected, as described previously [16].

### 2.4. Assessment of Nerve Fibers

Formalin fixed paraffin embedded (FFPE) blocks were retrieved from the archive of the local institute of pathology and slides were cut to perform immunohistochemistry staining with the neuronal marker PGP9.5. For this, we used sections (2.5 μm) deparaffinized in xylene and rehydrated in graded alcohols. The tissue was heated in citrate buffer (Agilent, Santa Clara, CA, USA) (pH 6.0) at 95–100 °C for 5 min and colled down for 20 min. The immunostaining anti-rabbit PGP9.5 (Dako antibody 1:100, (Agilent, Santa Clara, CA, USA)) was incubated overnight at 4 °C. All slides contained tumor tissue and the peritumoral region and were scanned with a Ventana digital slide scanner (Roche, Rotkreuz, Switzerland). A single digital image was uploaded in Qupath 0.1.6 (Centre for Cancer Research & Cell Biology at Queen’s University Belfast, United Kingdom). As previously described, nerve fiber count was analyzed by a trained pathologist who was blinded to the clinical outcomes of the individual patients. The presence of nerve fascicles at the invasive tumor margin with diameters of <100 μm was determined in 20 continuous visual fields at ×200 magnification by manual counting without the utilization of computer methods [16,19].

### 2.5. Statistical Analysis

The statistical endpoint of this study was cancer-specific survival (CSS), which was defined from the date of resection to the date of tumor-specific death. Deaths not associated with the tumor, e.g., cardiovascular events etc., were censored at the time of death. The secondary endpoint was recurrence-free survival (RFS), which was defined as the period from surgery to the date of first recurrence. Patients without tumor recurrence were censored at the time of death or at the last follow-up. Perioperative mortality was defined as in-hospital mortality. For NF categorization, a cut-off level was calculated by the receiver operating characteristic (ROC)-analysis of CCS with respect to NFs, as previously described [16,19]. Differences between the groups were evaluated by the Mann–Whitney-U-Test in case of continuous variables, while the chi-squared test, fisher’s exact test or linear-by-linear association in accordance with scale and number count were used in case of categorical variables. The associations of CSS and RFS with clinico-pathological variables were determined using univariate and multivariable Cox regression analyses in a backward selection model. Survival curves were generated by the Kaplan–Meier method and compared with the log-rank test. Median follow up was calculated with the reverse Kaplan–Meier method. *p*-values were given for two-sided testing and the level of significance was set to *p* < 0.05. All analyses were performed using SPSS Statistics 24 (IBM Corp., Armonk, NY, USA)).

## 3. Results

### 3.1. Patient Cohort

The patient cohort consisted of 55 women (57%) and 41 men (43%) with a median age of 65 years and the majority being assessed as ASA (American Society of Anesthesiologists classification) III or higher (53%, 51/96). Neoadjuvant therapy was applied in a small number of patients (8%, 8/96). Most of the patients underwent major liver surgery (66%, 64/96) to achieve R0 resections. Accordingly, an R0 status was observed in 93% (88/96) of the cohort. Further, nodal metastases were present in 30% of the patients (27/90). Major complications, as defined by Clavien–Dindo ≥ IIIa, were observed in 35% (34/96) of the cases. Patients displaying perioperative mortality were excluded from the analysis, as stated above. Further general demographic and clinico-pathological characteristics of the study cohort are presented in Table 1.

### 3.2. Group Categorization and Comparative Analysis with Respect to Nerve Fiber Density

A ROC analysis evaluating the total number of NFs (median number NFs in the cohort: 0 (range: 0–35)) for patients who survived at least 3 years versus patients who died during follow up was conducted. The corresponding area under the curve (AUC) was 0.618 (95% confidence interval (CI): 0.502–0.775). A cut-off value for NFs was determined with respect to optimized accuracy and equal weight for sensitivity and specificity errors (0 NF and ≥1 NF). Using the established cut-off value, the median CSS was 51 months in patients with NFs and 27 months in patients without NFs (*p* = 0.043 log rank).

A comparative group analysis regarding NFs was further carried out between patients with NFs (*n* = 45) and without NFs (*n* = 51). Here, no clinical differences were observed. Of note, no statistical differences in pathological characteristics, e.g., pT category (*p* = 0.400), pN category (*p* = 0.854), tumor grading (*p* = 0.114), lymphovascular invasion (LVI, *p* = 0.618), microvascular invasion (MVI, *p* = 0.252) and PNI (*p* = 0.175), were detected between the groups. However, the median CSS (51 months (95% CI: 12–90) vs. 27 months (95% CI: 19–35), *p* = 0.043 log rank) and the median RFS (20 months (95% CI: 0–41) vs. 8 months (95% CI: 5–11), *p* = 0.043 log rank) were significantly longer in patients with NFs compared to patients without NFs. A detailed overview of the cohort and both subgroups is outlined in Table 1.

### 3.3. Cox Regression Analysis

To investigate predictors of CSS and RFS in the overall cohort, univariate and multivariable Cox regressions were carried out. Here, in univariate analysis, postoperative complications (*p* = 0.007), N-category (*p* = 0.001), tumor grading (*p* = 0.014), LVI (*p* = 0.001), PNI (*p* = 0.011) and NFs (*p* = 0.048) were significantly associated with CSS. All variables showing *p*-value < 0.10 were included in a multivariable Cox regression model. Here, preoperative hemoglobin (hazard ratio (HR) = 0.51, *p* = 0.024), N-category (HR = 4.78, *p* = 0.001), NFs (HR = 0.47, *p* = 0.024) and neoadjuvant therapy (HR = 8.84, *p* = 0.002) were identified as independent predictors of CSS (Table 2).

In univariate analysis, postoperative complications (*p* = 0.028), intraoperative blood transfusions (*p* = 0.034), duration of hospitalization (*p* = 0.031), N-category (*p* = 0.001), microvascular invasion (MVI, *p* = 0.012), LVI (*p* = 0.013) and NFs (*p* = 0.009) showed significant associations with RFS. In the following multivariable Cox regression model, duration of hospitalization (HR = 1.78, *p* = 0.049), N-category (HR = 2.36, *p* = 0.010) and NFs (HR = 0.39, *p* = 0.002) were identified as independent predictors of RFS (Table 3).

### 3.4. Suvival Analysis

**Figure 3:**
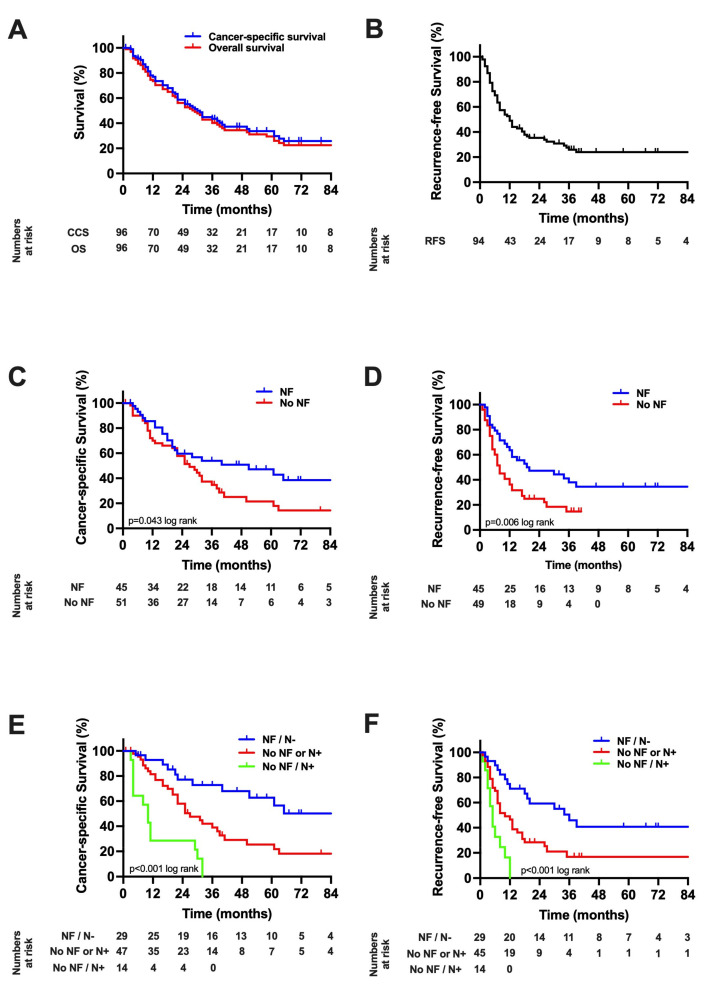
Oncological survival in intrahepatic cholangiocarcinoma (**A**): Cancer-specific and overall survival. The median CSS was 30 months and the median OS 28 months, respectively. (**B**): Recurrence-free survival. The median RFS was 12 months. (**C**): Cancer-specific survival stratified by nerve fibers. The median CSS was 51 months in patients with NF compared to 27 months (*p* = 0.043 log rank). (**D**): Recurrence-free survival stratified by nerve fibers. The median RFS was 20 months in patients with NF compared to 8 months in patients without NF (*p* = 0.006 log rank). (**E**): Cancer-specific survival stratified by nerve fibers and pN category. The mean CSS was 89 months in patients with NF and negative lymph nodes, while the median CCS was 27 months (95% CI: 38–64) in patients with either positive lymph nodes or without NF but not both, and 10 months in patients with both positive lymph nodes and low NF (*p* = 0.001 log rank). (**F**): Recurrence-free survival stratified by nerve fibers and pN category. The median RFS was 36 months in patients with NF and negative lymph nodes, 10 months (95% CI: 8–82) in patients with either positive lymph nodes or no NF but not both, and 5 months in patients with both positive lymph nodes and no NF (*p* = 0.001 log rank). CI, confidence interval; CSS, cancer-specific survival; RFS, recurrence-free survival; OS, overall survival.

After a median follow-up of 67 months, the median CSS of the whole cohort was 30 months (95% CI: 23–37), the median OS 28 months (95% CI: 20–36) and the median RFS 12 months (95% CI: 8–16, Figure 3A,B). A Kaplan–Meier analysis with respect to NFs showed a median CSS of 51 months (95% CI: 48–132, 3-year-CSS = 54%, 5-year-CSS = 47%) in patients with NFs compared to 27 months (95% CI: 19–47, 3-year-CSS = 35%, 5-year-CSS = 21%) in patients without NFs (*p* = 0.043 log rank, Figure 3C). Further, RFS was significantly lower in patients without NFs (8 months (95% CI: 5–11)) compared to patients with NFs (20 months (95% CI: 0–41), *p* = 0.006 log rank, Figure 3D). As both NF and N-category were independent predictors of CCS in the multivariable Cox regressions, a combined Kaplan–Meier analysis was conducted and showed a mean CSS of 89 months (95% CI: 65–111, 3-year-CSS = 73%, 5-year-CSS = 62%) in patients with NFs and negative lymph nodes, a median CCS of 27 months (95% CI: 17–37, 3-year-CSS = 36%, 5-year-CSS = 25%) in patients with either positive lymph nodes or no NFs but not both, and 10 months (95% CI: 6–14, 3-year-CSS = 0%, 5-year-CSS = 0%) in patients with both positive lymph nodes and no NFs (*p* = 0.001 log rank, Figure 3E). Accordingly, the median RFS was 36 months (95% CI: 24–48) in patients with NFs and negative lymph nodes, 10 months (95% CI: 6–15) in patients with either positive lymph nodes or no NFs but not both, and 5 months (95% CI: 3–6) in patients with both positive lymph nodes and no NFs (*p* = 0.001 log rank, Figure 3F).

### 3.5. Histological Characteristics

**Figure 4:**
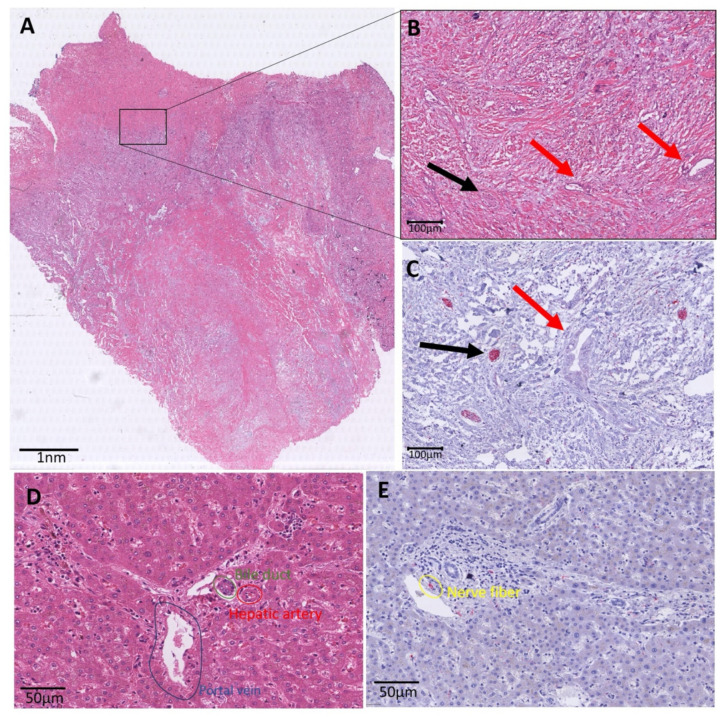
Histology overview of iCCA. (**A**): Whole slide H&E image of an iCCA. At the edge of the slide, normal liver parenchyma is displayed. More centrally, a lesion is shown with a rim of vital tumor cells, and centrally, a pale area corresponding with necrosis. (**B**): Zoomed in image of the black marked box in A. This H&E image shows small tumor glands in abundant stroma marked with red arrows. The black arrow points to a nerve fiber, which is not easily visible on this HE staining. (**C**): Black box area in the immunohistochemical PGP9.5 staining. This staining makes it easier to recognize the small nerve fibers (red in the PGP9.5 immunohistochemistry and marked with a black arrow). The red arrow is pointed at the tumor. (**D**): Zoomed in image of a portal tract. The portal tract illustrates the bile duct (marked in green), the hepatic artery (marked in red) and the portal vein (marked in blue). (**E**): Zoomed in image of a portal tract with PGP9.5 staining. In this zoomed in image, the small nerve fiber is nicely illustrated in red (positive immunohistochemical staining) and marked in yellow.

Scanned images of the H&E and PGP9.5 were descriptively analyzed. The region marked as tumor on the H&E staining was identified on the PGP immunostaining as well. Nerve fibers in the TME were observed and counted according to the previously described method. We observed that the small nerve fibers were mainly located at the periphery of the tumor and not in the center (Figure 4).

## 4. Discussion

ICCA is commonly diagnosed in advanced disease stages and associated with dismal oncological survival [1]. Despite recent advances in diagnosis, operative and systemic therapy, the 5-year-surival has not substantially improved and rarely reaches over 20%, with high rates of tumor recurrence being the main reason for these discouraging results [1,21,22]. Based on this, the identification of novel prognostic (bio)markers may provide insight into the underlying tumor biology of the disease and has the potential to further improve individualized medical management of these complex patients [15]. In this translational retrospective study, we identified NFs as a powerful novel prognostic biomarker for long-term oncological outcome in iCCA patients. Further, we could also demonstrate that the combination of NFs and traditional nodal status shows an excellent ability to stratify these patients regarding the overall prognosis after curative-intent liver resection.

NFs are believed to play an essential role in the crosstalk between tumor cells and other components of the TME such as immune cells or cancer-associated fibroblasts (CAF) [23,24,25,26]. This inter-cellular cross talk is partly based on neurotransmitters from NFs interacting with tumor cells, or ones that are released from cancer cells binding to receptors located on NFs [27,28,29,30]. Further, CAF-associated remodeling of the extracellular matrix does also result in neuron growth [23,24]. After the above-mentioned functions of NFs were described, their oncological role has been investigated in various malignancies, e.g., gastric and colorectal carcinomas [17,18]. Interestingly, in these disease entities, high density of NFs is associated with impaired oncological outcome in contrast to the observation in our present study. However, NFs seem to play a different role in cancer, depending on the tumor entity and, most probably, depending on the specific tumor microenvironment. Nonetheless, results from the present study are in line with our previous report regarding pCCA. Importantly, a recent report by Iwasaki et al. also identified a low density of NFs to be independently associated with reduced survival after surgical resection in PDAC [16,19,20]. The detailed underlying mechanistic explanation of this clinical observation is not yet clear and is beyond the scope of this clinical study. In former observations of our group, we have identified some of the small nerve fibers in the TME to be of parasympathetic origin [16]. Of note, these small NFs must be differentiated from larger preexisting nerve trunks, which can show tumor infiltration of the epineurial, perineural and endoneurial space of the nerve and are, therefore, traditionally used to define PNI [16,31,32]. Unfortunately, the role of the nervous system in cancer initiation and progression is not yet fully understood. However, some basic research does suggest some antitumor effects of the parasympathetic system, e.g., increased tumor growth and impaired survival in a vagotomized PDAC mouse model [33]. Another report of Kamiya et al. showed a decreased local tumor progression and attenuation of the development of distant metastases after increasing parasympathetic neurotransmission in a xenograft model of breast cancer [34]. While being speculative in nature, these findings might also provide a partial explanation for our clinical observations.

The identification of low-risk and high-risk cohorts and the selection of NF cutoffs appears to differ largely between various tumor entities. In breast cancer, NF density was categorized into no NFs, weak expression with 1–10 NFs and moderate/strong expression with >10 NFs by Zhao et al. [35]. In PDAC, intrapancreatic neural density was defined as low with ≤7 NFs and as high with >7 NFs [19]. In our previous work focusing on pCCA, we defined NF density as low in cases with <10 NFs and a high in individuals with ≥10 NFs [16]. While the differences of optimal cut-off values for risk stratification of different tumors and/or patient cohorts certainly appear logical from a statistical point-of-view and are frequently observed in case of other prognostic (bio)markers as well, the distinct observations made in this iCCA cohort (NFs vs. no NFs) and in our previous analysis regarding pCCA must be discussed critically [16]. PCCA are relatively homogeneous in their histological characteristics and are basically conventional mucin-producing adenocarcinomas or papillary tumors [36]. In contrast, iCCA can be divided into several histological subtypes with a conventional type, cholangiolocarcinomas and rare variants [37]. Conventional iCCA can be subdivided into large duct type and small duct type cancers. Large duct iCCA arises from large intrahepatic bile ducts and is histologically a mucin-producing tumor arranged in a large duct or papillary architecture, similarly to pCCA [38]. Small duct iCCA, in contrast, is a tubular or acinar adenocarcinoma with nodular growth and no or minimal mucin production, which originates from smaller intrahepatic bile ducts [39]. Notably, these histological differences also reflect the molecular heterogeneity of iCCA [37]. Small duct iCCA can often show isocitrate dehydrogenase (*IDH1*, *IDH2*) mutations or fibroblast growth factor receptor 2 (FGFR2) fusions [40,41]. In contrast, large duct iCCA shows a high frequency of mutations in Kirsten Rat Sarcoma (*KRAS*) and/or Tumor Protein 53 (*TP53*) genes similar to pCCA [42]. Given the similarities of one iCCA subtype with pCCA and the considerable differences of some iCCA subtypes in molecular and histological characteristics to pCCA, it might lead us to the assumption that the whole entity of iCCA might be strongly heterogeneous, which may require a distinct NF cut-off identification to stratify high- and low-risk patients in the future. Unfortunately, molecular data was not available for analysis in our cohort to correlate the NFs characteristics with separate genetic subtypes of iCCA.

From a clinical-oncological point of view, our results are in line with previous findings. The analyzed cohort of iCCA patients showed a 5-year-CCS of 34% and 5-year-OS of 29%. In a systematic review of 57 studies including more than 4500 patients undergoing liver resection for iCCA, 5-year-OS ranged from 5% to 56% depending on the frequency of typical risk factors, e.g., age, pathological characteristics or lymph node metastases [43]. In our cohort, absence of NFs, nodal metastases, low preoperative hemoglobin and neoadjuvant therapy were independent predictors of inferior CCS. In fact, neoadjuvant therapy (HR = 8.84) had the most pronounced impact on survival followed by lymph node metastases (HR = 4.78) and NFs (HR = 0.47). The role of neoadjuvant therapy in iCCA is a matter of an ongoing debate and its role remains to be defined. In a large multicentric analysis, no difference in oncological survival between patients undergoing upfront surgery versus patients who underwent preoperative chemotherapy was observed; however, patients scheduled for neoadjuvant therapy displayed significantly more advanced tumors in this study [44]. This reflects common international standards and also the clinical routine in our centre proceeding with upfront surgery in patients with resectable disease [1,45]. Neoadjuvant therapy in our study cohort was, therefore, applied to a small subset of patients (*n* = 8) presenting with a large tumor mass and/or major vascular involvement. The biased selection of this high-risk subgroup of patients does certainly explain the high hazard ratio in our analysis.

The presence of NFs, in particular, resulted in a prominent division of our cohort according to long-term outcome, with a median CCS of 51 months (5-year-CSS = 47%) in patients with NFs in the TME and a median CCS of 27 months (5-year-CSS = 21%) in patients without NFs in the TME, indicating an important predictive value of this histological marker. This large difference in survival can be attributed to the good predictive value of NFs in terms of tumor recurrence. Here, the absence of NFs among lymph node metastases and long duration of hospitalization were associated with inferior RFS. The median RFS was 20 months (3-year-RFS 38%) in patients with NFs and 8 months (3-year-RFS 15%) in patients without NFs. This well-illustrates that tumor recurrence remains the major problem in iCCA patients. Repeated liver resections which provide appropriate long-term outcome are unfortunately only applicable for the minority of patients experiencing tumor recurrence [46,47]. Most patients still undergo systemic therapy, which is characterized by limited response and resistance to chemotherapy, thus resulting in early disease progression [6].

The prognostic role of nodal status in iCCA is abundantly discussed elsewhere [15,48,49]. As nodal status was not associated with the presence of NFs in the TME in our group comparison (Table 1) and both variables showed significant risks in our multivariable Cox regression models for RFS and CCS, we created subgroups based on NFs and the pN category to stratify patients regarding long-term outcome. Here, patients with low oncological risk (presence of NFs and negative lymph nodes) displayed a compelling outcome with a 5-year-CCS of 62%, while patients with medium risk (absence of NFs or positive lymph nodes but not both) showed an “average” outcome with a 5-year-CCS of 25%, followed by patients with high risk (absence of NFs and positive lymph nodes) yielding a dismal long-term outcome with a 3-year-CCS of 0%. This observation is novel and interesting with a potential impact on the clinical management of these patients. Surgical resection in patients with positive lymph nodes alone is usually associated with inferior outcome and the survival benefit appears marginally to systemic therapy alone in some previous reports [50,51]. Therefore, some authors suggest a very critical approach to surgery in iCCA with nodal metastases [1]. Our department strategy comprises a radical approach to iCCA that does not deny patients the possibility of radical surgery even in cases where lymph node positivity is confirmed in intraoperative frozen sections [7,15]. However, it has to be acknowledged that our high-risk subgroup (absence of NFs and positive lymph nodes) showed a median CCS of 10 months, which is indeed inferior to the results of systemic therapy, as shown in the ABC-02 trial evaluating Gemcitabine and Cisplatin in the palliative setting displaying a median OS of 12 months [6]. As iCCA does usually present with a notable tumor mass at the time of diagnosis, tissue for preoperative histological analysis is more easily obtainable by biopsy compared to pCCA [16]. If the absence NFs is, therefore, known preoperatively and other oncological risk factors are present in the individual patient, e.g., positive lymph nodes determined by preoperative endoscopic ultrasound-guided fine-needle biopsy, staging laparoscopy or by intraoperative frozen sections, liver resection should be carefully evaluated in these patients as the oncological benefit might not justify the perioperative risks of surgery. This statement represents one of the most important clinical messages of this study.

As with other retrospective translational studies, our analysis has some potential limitations. First, all patients of this study underwent surgery in a single center in accordance with the authors’ individual approach to iCCA, and all clinical data were obtained in a retrospective fashion. Second, the retrospective nature of our study resulted in some missing data, which would have been interesting to report in the context of the oncological analysis, e.g., CA19-9. Third, the limited sample size did not allow the division of the cohort into a training and validation set, which would have strengthened our statistical analysis. Further, our data and the resulting observations would certainly require confirmation within a large independent and, optimally, multi-center dataset to reduce the risk of bias, and no amount of reanalysis of the current cohort can eliminate this need. Fourth, it is not possible to deeply investigate the associations of NFs with tumor characteristics using our data. Such investigations focusing on the underlying pathophysiological mechanisms may include extensive radiologic and biological data (e.g., tumor genetics) which were not available for this study but should be the topic of further investigations.

## 5. Conclusions

Notwithstanding the above-mentioned limitations, we have identified NFs as a novel and important prognostic biomarker in iCCA patients. The presence of NFs alone and in combination with nodal status allow for stratification of iCCA patients in terms of oncological outcome after curative-intent surgery. These findings have the potential to be clinically implemented since the NF count requires only one simple immunohistochemical staining and the nodal status is a routine characteristic in the pathology report. Larger, multi-center studies are needed to confirm and validate our findings.

## Figures and Tables

**Table 1:** Patients’ characteristics. Data presented as median and interquartile range if not noted otherwise. ALT, alanine aminotransferase; ASA, American society of anesthesiologists classification; AST, aspartate aminotransferase; BMI, body mass index; CSS, cancer-specific survival; GGT, gamma glutamyltransferase; INR, international normalized ratio; LVI, lympho-vascular invasion; MVI, microvascular invasion; NF, nerve fibers; PNI, perineural invasion, RFS, disease-free survival.

Demographics	Overall Cohort (*n* = 96)	NF Positive (*n* = 45)	NF Negative (*n* = 51)	*p* Value
Gender, m/f (%)	41 (43)/55 (57)	18 (40)/27 (60)	23 (45)/28 (55)	0.614
Age (years)	65 (58–73)	66 (60–75)	62 (56–72)	0.330
BMI (kg/m^2^)	25 (22–29)	25 (22–29)	25 (23–29)	0.733
ASA, n (%)				0.230
I	3 (3)	1 (2)	2 (4)	
II	42 (44)	21 (47)	21 (41)	
III	48 (50)	20 (44)	28 (55)	
IV	3 (3)	3 (7)	0	
V	0	0	0	
**Clinical chemistry**				
Albumin (g/dL)	44 (41–46)	43 (40–46)	44 (41–46)	0.314
AST (U/L)	34 (26–47)	32 (24–44)	37 (29–53)	0.077
ALT (U/L)	29 (20–53)	25 (17–50)	31 (22–57)	0.169
GGT (U/L)	114 (65–304)	88 (65–501)	118 (62–265)	0.876
Total bilirubin (mg/dL)	0.5 (0.4–0.7)	0.5 (0.4–0.7)	0.6 (0.4–0.8)	0.117
Platelet count (/nL)	245 (197–307)	251 (194–303)	238 (198–315)	0.925
Alkaline Phosphatase (U/L)	117 (90–258)	108 (78–301)	125 (91–246)	0.628
Prothrombin time (%)	100 (95–109)	100 (96–111)	102 (94–108)	0.942
INR	0.98 (0.95–1.03)	0.97 (0.93–1.03)	0.99 (0.96–1.03)	0.386
Hemoglobin (g/dL)	13 (12–14)	13 (12–14)	13 (12–14)	0.248
**Operative Data**				
Operative time (minutes)	285 (221–345)	285 (227–338)	285 (212–345)	0.895
Operative procedure, *n* (%)				0.410
Monosegmentectomy/atypical	9 (9)	6 (13)	3 (6)	
Bisegmentectomy	7 (7)	4 (9)	3 (6)	
Right/left hepatectomy	31 (32)	17 (38)	14 (28)	
Ext. right/left hepatectomy	20 (21)	7 (16)	13 (26)	
Right/left trisectionectomy	13 (14)	4 (9)	9 (18)	
Others	16 (17)	7 (16)	9 (18)	
Intraoperative blood transfusion	0 (0–2)	0 (0–2)	0 (0–2)	0.750
**Pathological examination**				
R0 resection, *n* (%)	88 (93)	41 (93)	47 (92)	0.849
pT category, *n* (%)				0.400
1	38 (40)	23 (51)	15 (29)	
2	41 (42)	15 (34)	26 (51)	
3	12 (13)	5 (11)	7 (14)	
4	5 (5)	2 (4)	3 (6)	
pN category				0.854
N0	63 (70)	29 (69)	34 (71)	
N1	27 (30)	13 (31)	14 (29)	
Tumor grading, *n* (%)				0.114
G1	0	0	0	
G2	66 (76)	35 (83)	31 (69)	
G3	21 (24)	7 (17)	14 (31)	
G4	0	0	0	
MVI, *n* (%)	32 (35)	12 (28.6)	20 (40)	0.252
LVI, *n* (%)	17 (19)	7 (17)	10 (21)	0.618
PNI, *n* (%)	22 (46)	11 (58)	11 (38)	0.175
**Postoperative Data**				
Intensive care, days	1 (1–2)	1 (1–1)	1 (1–2)	0.114
Hospitalization, days	13 (8–24)	14 (8–26)	12 (8–22)	0.769
Postoperative complications, *n* (%)				0.898
No complications	36 (38)	15 (33)	21 (41)	
Clavien–Dindo I	2 (2)	1 (2)	1 (2)	
Clavien–Dindo II	24 (25)	13 (29)	11 (22)	
Clavien–Dindo IIIa	19 (20)	10 (22)	9 (18)	
Clavien–Dindo IIIb	9 (9)	4 (9)	5 (10)	
Clavien–Dindo IVa	6 (6)	2 (4)	4 (8)	
Clavien–Dindo IVb	0	0	0	
Clavien–Dindo V	0	0	0	
**Oncologic Data**				
Adjuvant therapy	30 (31)	10 (22)	20 (39)	0.073
Neoadjuvant therapy, *n* (%)	8 (8)	3 (7)	5 (10)	0.579
Median RFS, months (95% CI)	12 (8–16)	20 (0–41)	8 (5–11)	**0.006**
Median CSS, months (95% CI)	30 (23–37)	51 (12–90)	27 (19–35)	**0.043**

**Table 2:** Univariate and multivariable analysis of cancer-specific survival in intrahepatic cholangiocarcinoma. Various parameters are associated with cancer-specific survival. ALT, alanine aminotransferase; ASA, American society of anesthesiologists classification; AST, aspartate aminotransferase; BMI, body mass index; GGT, gamma glutamyltransferase; INR, international normalized ratio; LVI, lympho-vascular invasion; MVI, microvascular invasion; NF, nerve fibers; PNI, perineural invasion.

	Univariate Analysis		Multivariable Analysis	
	HR (95% CI)	*p* Value	HR (95% CI)	*p* Value
**Demographics**				
Sex (male = 1)	0.76 (0.45–1.27)	0.297		
Age (≤65 years = 1)	1.30 (0.77–2.15)	0.330		
BMI (≤25 kg/m^2^ = 1)	1.17 (0.67–1.96)	0.549		
ASA (I/II = 1)	1.31 (0.79–2.19)	0.299		
**Clinical chemistry**				
Albumin (≤45 g/L = 1)	0.86 (0.52–1.43)	0.560		
AST (≤35 U/L = 1)	1.15 (0.69–1.93)	0.588		
ALT (≤30 U/L = 1)	1.38 (0.82–2.35)	0.228		
GGT (≤120 U/L = 1)	1.39 (0.83–2.34)	0.214		
Bilirubin (≤0.5 mg/dL = 1)	1.53 (0.91–2.57)	0.105		
Alkaline phosphatase (≤115 U/L = 1)	1.69 (0.99–2.89)	0.054	excluded	
Platelet count (≤250/nL = 1)	0.82 (0.49–1.37)	0.445		
INR (≤1 = 1)	1.54 (0.91–2.61)	0.107		
Hemoglobin (≤13 g/dL = 1)	0.61 (0.37–1.03)	0.063	0.51 (0.27–0.93)	**0.024**
**Operative data**				
Operative time (≤300 min = 1)	1.11 (0.68–1.80)	0.682		
Type of hepatectomy		0.935		
Right/left hepatectomy	1			
Others	0.92 (0.541–1.62)			
Blood transfusion (no = 1)	1.52 (0.91–2.57)	0.113		
**Postoperative data**				
Clavien–Dindo Score (CD I/II = 1)	2.05 (1.22–3.47)	**0.007**	exlcuded	
Intensive care (≤1 day = 1)	1.49 (0.85–2.62)	0.168		
Hospitalization (≤13 days = 1)	1.52 (0.91–2.54)	0.106		
**Pathological data**				
R1 resection (no = 1)	1.58 (0.63–3.96)	0.329		
pT category (T1/T2 = 1)	1.49 (0.81–2.77)	0.203		
pN category (N0 = 1)	4.32 (2.48–7.52)	**0.001**	4.78 (2.54–9.01)	**0.001**
Tumor grading (G1/G2 = 1)	2.13 (1.16–3.89)	**0.014**	excluded	
MVI (no = 1)	1.59 (0.95–2.68)	0.078	excluded	
LVI (no = 1)	3.60 (1.92–6.76)	**0.001**	excluded	
PNI (no = 1)	2.49 (1.23–5.01)	**0.011**		
NF (no = 1)	0.58 (0.34–0.99)	**0.048**	0.47 (0.24–0.90)	**0.024**
**Oncological data**				
Neoadjuvant therapy (no = 1)	2.18 (0.87–5.50)	0.098	8.84 (2.20–35.49)	**0.002**
Adjuvant therapy (no = 1)	1.17 (0.67–2.04)	0.587		

**Table 3:** Univariate and multivariable analysis of recurrence-free survival in intrahepatic cholangiocarcinoma. Various parameters are associated with recurrence-free survival. ALT, alanine aminotransferase; ASA, American society of anesthesiologists classification; AST, aspartate aminotransferase; BMI, body mass index; GGT, gamma glutamyltransferase; INR, international normalized ratio; LVI, lympho-vascular invasion; MVI, microvascular invasion; NF, nerve fibers; PNI, perineural invasion.

	Univariate Analysis		Multivariable Analysis	
	HR (95% CI)	*p* Value	HR (95% CI)	*p* Value
**Demographics**				
Sex (male = 1)	1.00 (0.60–1.66)	0.993		
Age (≤65 years = 1)	0.92 (0.56–1.52)	0.755		
BMI (≤25 kg/m^2^ = 1)	0.84 (0.51–1.38)	0.486		
ASA (I/II = 1)	1.25 (0.76–2.05)	0.383		
**Clinical chemistry**				
Albumin (≤45 g/L = 1)	0.96 (0.59–1.58)	0.872		
AST (≤35 U/L = 1)	0.97 (0.59–1.60)	0.916		
ALT (≤30 U/L = 1)	1.34 (0.81–2.24)	0.258		
GGT (≤120 U/L = 1)	1.47 (0.89–2.45)	0.137		
Bilirubin (≤0.5 mg/dL = 1)	1.31 (0.79–2.17)	0.302		
Alkaline phosphatase (≤115 U/L = 1)				
Platelet count (≤250/nL = 1)	0.79 (0.48–1.32)	0.373		
INR (≤1 = 1)	1.56 (0.92–2.65)	0.099	excluded	
Hemoglobin (≤13 g/dL = 1)	0.67 (0.40–1.10)	0.112		
**Operative data**				
Operative time (≤300 min = 1)	1.05 (0.64–1.74)	0.837		
Type of hepatectomy		0.538		
Right/left hepatectomy	1			
Others	1.18 (0.68–2.03)			
Blood transfusion (no = 1)	1.74 (1.04–2.90)	**0.034**	excluded	
**Postoperative data**				
Clavien–Dindo Score (CD I/II = 1)	1.78 (1.06–2.99)	**0.028**	excluded	
Intensive care (≤1 day = 1)	1.27 (0.73–2.21)	0.410		
Hospitalization (≤13 days = 1)	1.73 (1.05–2.85)	**0.031**	1.78 (1.00–3.15)	**0.049**
**Pathological data**				
R1 resection (no = 1)	1.62 (0.64–4.07)	0.310		
pT category (T1/T2 = 1)	0.98 (0.48–1.98)	0.943		
pN category (N0 = 1)	2.84 (1.61–5.03)	**0.001**	2.36 (1.23–4.52)	**0.010**
Tumor grading (G1/G2 = 1)	1.32 (0.71–2.47)	0.386		
MVI (no = 1)	1.93 (1.15–3.21)	**0.012**	excluded	
LVI (no = 1)	2.22 (1.19–4.17)	**0.013**	excluded	
PNI (no = 1)	1.38 (0.69–2.87)	0.382		
NF (no = 1)	1.98 (1.19–3.31)	**0.009**	0.39 (0.21–0.71)	**0.002**
**Oncological data**				
Neoadjuvant therapy (no = 1)	1.96 (0.84–4.60)	0.121		
Adjuvant therapy (no = 1)	1.15 (0.68–1.95)	0.597		

## Data Availability

The data presented in this study are available on request from the corresponding author.
